# Pie-like electrode design for high-energy density lithium–sulfur batteries

**DOI:** 10.1038/ncomms9850

**Published:** 2015-11-26

**Authors:** Zhen Li, Jin Tao Zhang, Yu Ming Chen, Ju Li, Xiong Wen (David) Lou

**Affiliations:** 1 School of Chemical and Biomedical Engineering, Nanyang Technological University, 62 Nanyang Drive, Singapore 637459, Singapore; 2 Department of Nuclear Science and Engineering, Massachusetts Institute of Technology, Cambridge, Massachusetts 02139, USA; 3 Department of Materials Science and Engineering, Massachusetts Institute of Technology, Cambridge, Massachusetts 02139, USA

## Abstract

Owing to the overwhelming advantage in energy density, lithium–sulfur (Li–S) battery is a promising next-generation electrochemical energy storage system. Despite many efforts in pursuing long cycle life, relatively little emphasis has been placed on increasing the areal energy density. Herein, we have designed and developed a ‘pie' structured electrode, which provides an excellent balance between gravimetric and areal energy densities. Combining lotus root-like multichannel carbon nanofibers ‘filling' and amino-functionalized graphene ‘crust', the free-standing paper electrode (S mass loading: 3.6 mg cm^−2^) delivers high specific capacity of 1,314 mAh g^−1^ (4.7 mAh cm^−2^) at 0.1 C (0.6 mA cm^−2^) accompanied with good cycling stability. Moreover, the areal capacity can be further boosted to more than 8 mAh cm^−2^ by stacking three layers of paper electrodes with S mass loading of 10.8 mg cm^−2^.

## Abstract (web summary)

Lithium-sulfur batteries are a promising candidate for next-generation battery technologies. Here, the authors report a pie-like structured electrode in which sulfur is confined in multichannel carbon nanofibers which is then coated by amino-functionalized graphene, leading to good balance between electrochemical performance and cell energy density.

## Graphical abstract

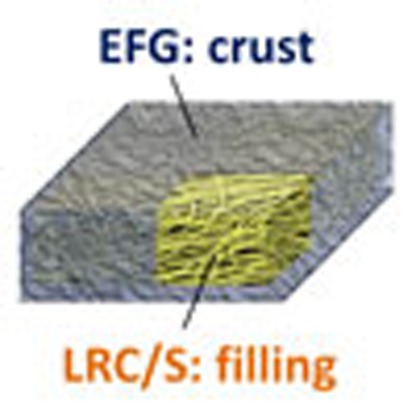

Although lithium ion batteries (LIBs) have achieved remarkable success in consumer electronics market in the past 20 years, traditional cathode materials based on the lithiated transition-metal oxide and phosphate are unable to satisfy the requirements of fast-developing portable electronics with ever-more interesting shape factors and flexibility requirements. What's more, many researchers believe that the energy density of perfectly developed LIBs still cannot meet the demands of electric vehicles and large-scale energy storage^1^,^2^. Lithium–sulfur (Li–S) batteries, an old electrochemical system first invented in 1960s^3^, have now been considered as a promising candidate for the next-generation batteries and attracted lots of attention in recent years, owing to their overwhelming advantage in gravimetric energy density. With a theoretical capacity and energy density of 1,675 mAh g^−1^ and 2,600 Wh kg^−1^, respectively, the practical gravimetric energy density of Li–S batteries is estimated to be 2–3 times higher than that of state-of-the-art LIBs^4^,^5^. In addition, the high natural abundance, low cost and environmental friendliness of sulfur make Li–S batteries attractive compared to current LIBs. However, the commercialization of rechargeable Li–S batteries is still hindered by several obstacles, including the low sulfur utilization and poor cycle life. These problems are mainly related to the insulating nature of sulfur, and the dissolution of long-chain polysulfides generated during cycling. To address these issues, various strategies have been developed to enhance the conductivity of the active material and trap the polysulfides within the cathode side, such as investigating new electrolytes^6^,^7^,^8^, modifying the separator^9^,^10^,^11^,^12^, protecting the lithium anode^13^,^14^ and inserting polysulfides-blocking interlayers^15^,^16^. Among them, most attentions have been paid to advancing the sulfur-based cathodes^17^. Remarkable progresses on sulfur-based cathodes have been made in recent years, spanning from nanocomposites with nanoporous carbon^18^,^19^,^20^,^21^,^22^,^23^, surface coating^24^,^25^,^26^,^27^,^28^, new binders^29^,^30^,^31^, using polar materials instead of nonpolar ones as the sulfur hosts^32^,^33^,^34^ and optimizing charge–discharge depth^35^ and so on. To date, the cycle life of Li–S batteries has reached more than 1,000 cycles^25^,^33^,^36^, and a cycle life of even more than 4,000 cycles has been obtained^37^.

Although it is undeniable that the cycle number is a critical parameter for the evaluation of batteries, a high-energy density is always the very initial motivation and the prime advantage of Li–S batteries^1^,^38^. However, the high specific gravimetric capacity and/or long cycle life cells in the literature are usually associated with low sulfur percentage in the cathode material (<70 wt%) and/or low sulfur areal mass loading in the composite electrode (<2 mg cm^−2^), making them less attractive for adoption in practical batteries. Therefore, it is necessary to develop new structures and materials that possess good cycling stability without sacrificing the areal energy density. Some new structured electrodes with high sulfur loading have been explored, such as graphene foam-based electrode^39^, carbon fibres cloth-based electrode^40^, layer-by-layer structured electrode^41^, free-standing carbon nanotube (CNT) paper-S electrode^42^ and thick slurry-coated carbon/sulfur composite electrode^43^, and many others. These works have promoted the areal capacity and energy density of Li–S batteries to a much higher level. But still, researches on high mass loading Li–S batteries with long cycle life are very challenging at this stage.

It is believed that free-standing carbon–sulfur composite ‘paper' electrodes are a great option in achieving high sulfur content, since they need not include additional binders, conductive carbon black and current collector in the overall cells, which occupy considerable mass and volume fractions of the traditional slurry coating–derived electrodes^38^. It is noted that one-dimensional nanomaterials are widely applied to form the free-standing electrodes in the fields of energy storage and conversion in the view of their unique low-dimensional properties^44^. Compared with traditional template methods, the electrospinning technique has much easier accessibility for mass production of 1D nanofibers with high surface-to-volume ratio^45^. For example, Yu's group prepared several electrospun-derived free-standing porous carbon nanofibers-based paper electrodes loaded with selenium and sulfur for lithium and sodium storage, demonstrating an efficient way to increase the active materials content without sacrificing the capacity utilization^46^,^47^.

The concept of interlayers introduced by Manthiram is a noteworthy technique in Li–S batteries for localizing polysulfides^15^. The insertion of a carbon-based film between cathode and separator can effectively restrict the dissolved polysulfides at the cathode side, and significantly improve the cycling stability. However, the attached interlayer decreases the sulfur content in the cell. In other words, with the extra mass of the second carbon layer, the areal energy density of a Li–S cell could be significantly reduced compared with melt-in carbon/sulfur composites^48^. Thus, it remains a great challenge to reduce the content of interlayer materials in the Li–S batteries to achieve high-energy density.

Herein, inspired by the structure of pie, we have designed and developed a free-standing pie-like paper electrode. Sulfur is confined in the three-dimensional (3D) interconnected lotus root-like multichannel carbon (LRC) nanofibers (LRC/S electrode; the ‘filling'), and a thin layer of ethylenediamine (EDA)-functionalized reduced graphene oxide (EFG) is coated on the surface of the LRC/S electrode (the ‘crust'). This design has multiple advantages. Specifically, each LRC/S nanofiber has multiple channels with average diameter of 60 nm, which provide large void space for sulfur accommodation, giving high mass loading of active material. Second, highly parallel channel walls inside each LRC nanofiber ensure close contact between carbon and sulfur/lithium sulfides. This forms an excellent conducting framework inside every single nanofiber, and leads to high utilization efficiency of the active sulfur material. Third, the unique design of the whole electrode, which is a 3D interconnected conductive framework of LRC nanofibers, reduces the resistance of electron and ion transport during the electrochemical redox processes, giving rise to high-discharge capacity. In addition, the thin EFG layer not only enhances the conductivity of the total electrode, but also effectively suppresses the diffusion of polysulfides at the cathode side, therefore, maintaining excellent cycling stability^49^. Moreover, the EFG ‘crust', which is only a small portion of the ‘pie', does not significantly affect the final gravimetric energy density of the electrode. With this rational design, the sulfur content of the LRC/S@EFG electrode reaches to 72.3 wt%, and the areal mass loading of sulfur is as high as 3.6 mg cm^−2^. Both of these two important energy density-related parameters are higher than most of reported literatures^2^, while the electrode is still able to deliver a high reversible specific capacity of 1,314 mAh g^−1^ (4.7 mAh cm^−2^) at 0.1 C (0.6 mA cm^−2^). Remarkably, the LRC/S@EFG electrode delivers discharge capacities of 1,215 mAh g^−1^ for the first cycle and 950 mAh g^−1^ for the 200th cycle at 0.2 C, corresponding to an extremely low capacity decay rate of 0.1% per cycle. The areal capacity can be further boosted to more than 6 or 8 mAh cm^−2^ by stacking two or three layers of LRC/S@EFG electrode. We believe that this design of pie-like electrode can also be applied in other energy conversion and storage systems.

## Results

### Synthesis and characterizations of the LRC/S@EFG electrode

**Figure 1:**
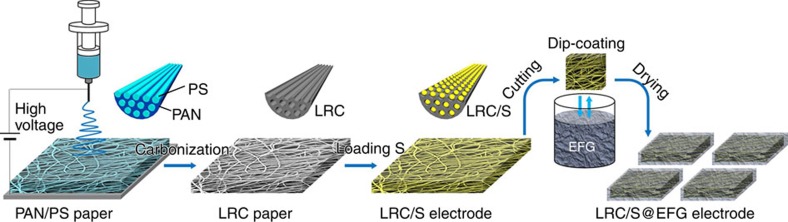
Illustration of the synthesis process of the LRC/S@EFG electrode. Electrospinning of precursor nanofibers of PAN/PS, followed by carbonization to form the LRC paper. After being loaded with sulfur, LRC/S electrodes are dip-coated with EFG.

Schematic of the synthesis process of LRC/S@EFG is shown in Fig. 1. In a typical procedure, a precursor of polyacrylonitrile (PAN) and polystyrene (PS) in N,N-dimethylformamide is fabricated into nanofiber web by electrospinning. After the carbonization process at 800 °C in argon atmosphere, PS is decomposed, and parallel channels are generated within the shells of carbonized PAN. Then, sulfur is introduced into the LRC channels by heating the commercial sulfur powder and LRC paper together in a sealed vessel at 300 °C for 12 h. After that, the free-standing LRC/S electrode is cut into smaller square pieces, and dip-coated with a layer of interconnected EFG nanosheets on their surfaces. When completely dried, the binder-free LRC/S@EFG electrodes are directly used as the working cathodes for electrochemical measurements.

**Figure 2:**
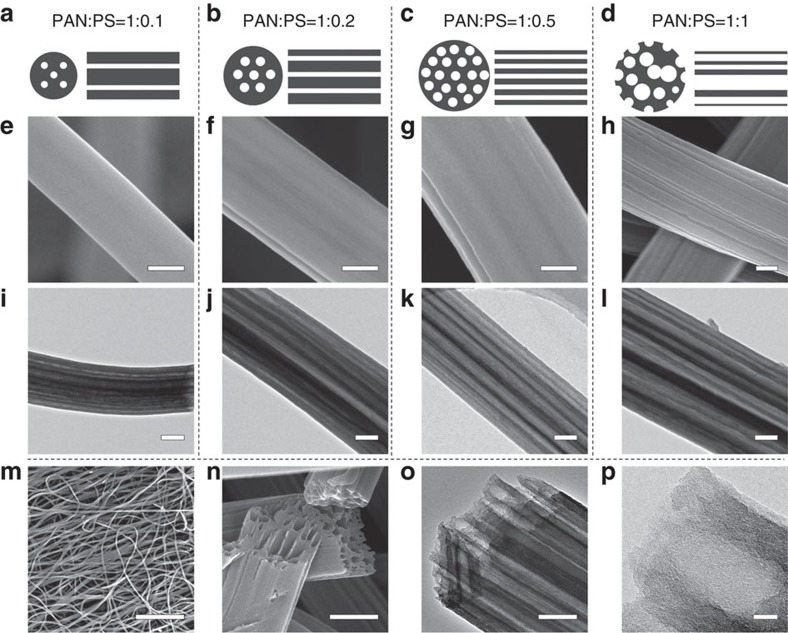
Characterizations of LRC nanofibers. (**a**–**d**) Schematic diagrams, (**e**–**h**,**m**,**n**) FESEM and (**i**–**l**,**o**,**p**) TEM images of LRC nanofibers based on various PAN/PS weight ratio: (**a**,**e**,**i**) 1:0.1, (**b**,**f**,**j**) 1:0.2, (**c**,**g**,**k**,**m**–**p**) 1:0.5, (**d**,**h**,**l**) 1:1. Scale bars, 200 nm (**e**–**l**,**o**), 20 μm (**m**), 500 nm (**n**), 20 nm (**p**).

We have developed a facile and scalable approach to synthesize free-standing LRC nanofibers mat by pyrolysis of electrospun PAN/PS nanofibers. It is found that when PS is added into the PAN solution, it forms a micro emulsion that could be stretched into nanoscaled wires in the PAN fibres during the electrospinning process, and then decomposed to generate nanochannels during the pyrolysis process (Supplementary Fig. 1). Transmission electron microscopy (TEM) characterizations demonstrate that there are many highly parallel channels in each nanofiber with no breaks (Fig. 2i–l). The channel structures of LRC nanofibers can be easily controlled by changing the weight ratio of PAN and PS from 1:0.1 to 1:1 (Fig. 2a–l). With the increase of the PS content, both channel diameters and channel numbers inside each LRC nanofiber increase accordingly, giving rise to an increase of the void space in LRC nanofibers (Fig. 2i–k). However, an ideal carbon structure should not only provide large internal space for high sulfur loading, but also have a firm shell for restricting the dissolution of polysulfides during cycling^50^,^51^. With the PAN/PS ratio of 1:1, there are many semicircular channels appearing on the surface of carbon nanofibers, and the dimensions of channels become non-uniform (Fig. 2h,l). Although it has the largest internal void space, the incomplete shell may increase the amount of exposed sulfur in the electrode, and lead to serious dissolution of polysulfides. With the PAN/PS ratio of 1:0.5, the surface of LRC nanofibers is smooth, and the inner channels are uniformly separated by thin carbon walls (Fig. 2g,k, Supplementary Fig. 2). Hence, all LRC nanofibers used in this work are fabricated according to the optimized PAN/PS ratio of 1:0.5.

The as-prepared LRC nanofibers interconnect with each other (Fig. 2m) forming a conductive mat with submicron/micron-sized interfiber porosity, which is beneficial for the penetration of electrolyte and fast ion transport. The lotus root-like multichannel structure can be directly observed from the cross section view of LRC nanofibers (Fig. 2n,o). High-resolution TEM observation further reveals that the shells of these lotus root-like nanofibers contain plenty of micropores (Fig. 2p). The microporous structure of channel walls is anticipated to play two important roles: (1) sulfur molecules could enter into the inner void space of LRC nanofibers through the micropores on the wall during the sulfur–carbon compositing process^23^,^37^; (2) during the cycling process, the microporous channel walls are efficient physical barriers for preventing the dissolution of polysulfides^23^,^52^. The nitrogen sorption measurement of LRC nanofibers reveals a porous structure with a specific surface area of 541 m^2^ g^−1^ (Supplementary Fig. 3a). We also directly measure the channel size of many LRC nanofibers from TEM images and the average channel size is determined to be ∼60 nm (Supplementary Fig. 3b).

**Figure 3:**
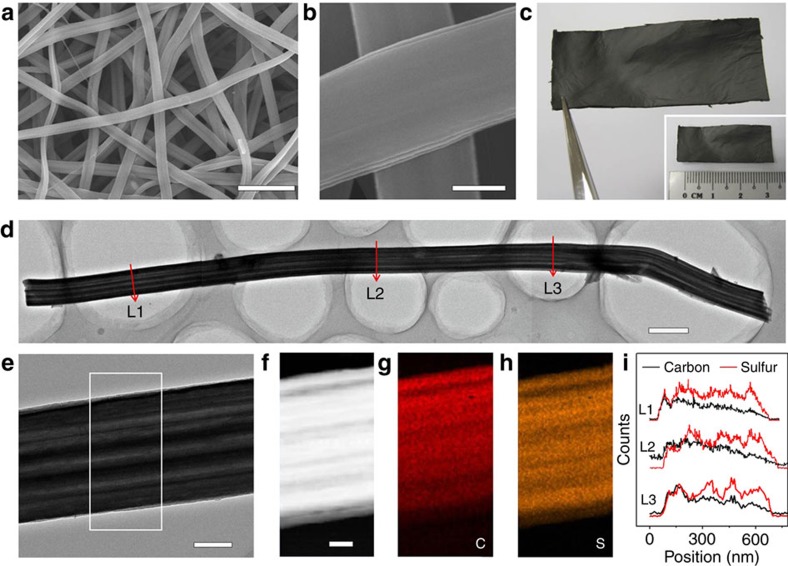
Characterizations of the LRC/S composite. (**a**,**b**) FESEM images, (**c**) digital photos, (**d**,**e**) TEM images, (**f**) dark-field TEM image and corresponding elemental mappings of (**g**) carbon and (**h**) sulfur of the LRC/S composite. (**i**) Linear EDX element distributions of carbon and sulfur along the arrow lines of L1–L3 on (**d**). Scale bars, 5 μm (**a**), 500 nm (**b**), 1 μm (**d**), 200 nm (**e**), 100 nm (**f**).

Since both pyrolytic carbon and sulfur are hydrophobic materials, it is easy for LRC frameworks to absorb sulfur molecules within the porous channels. The formed LRC/S electrode shows similar morphology and structure compared with the pristine LRC textile (Fig. 3a). The energy dispersive X-ray spectroscopy and X-ray diffraction results prove the presence of sulfur (Supplementary Figs 4 and 5), while the smooth surface of LRC/S nanofibers reveals that no additional sulfur particles exist outside the LRC nanofibers (Fig. 3b). Benefitting from the entangled network formed by LRC nanofibers, the LRC/S electrode retains the free-standing feature (Fig. 3c). To further investigate the sulfur distribution in LRC nanofibers, we compare the linear distributions of sulfur and carbon at three different positions on a single LRC/S composite nanofiber (Fig. 3d). All three linear scans display similar results (Fig. 3i), confirming the successful impregnation of sulfur in the whole multichannels inside the LRC nanofibers. TEM elemental mappings on a segment (Fig. 3e–h) and scanning electron microscope elemental mappings on several nanofibers (Supplementary Fig. 6) of LRC/S further confirm that sulfur is homogeneously distributed in the framework of the LRC nanofibers, with almost no sulfur particles adhering on the external surfaces.

**Figure 4:**
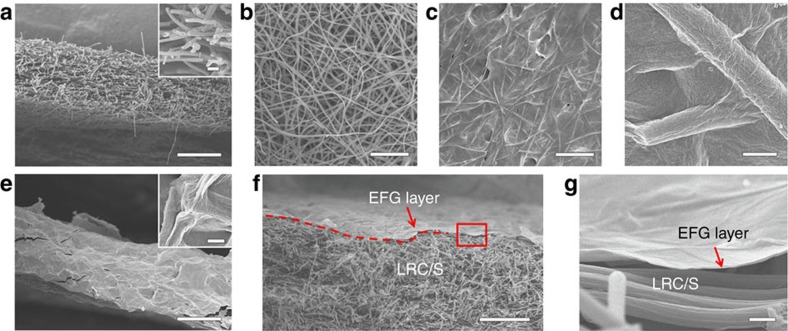
Characterizations of the LRC/S and LRC/S@EFG electrodes. FESEM images of (**a**,**b**) the LRC/S electrode, (**c**–**e**) the pie-like LRC/S@EFG electrode. (**f**,**g**) Cross section SEM image of a dissected LRC/S@EFG electrode. Scale bars, 50 μm (**a**,**e**,**f**), 20 μm (**b**,**c**), 1 μm ((**d**,**g**) inset of **e**), 2 μm (inset of **a**).

Although the LRC/S composite nanofibers have an ultrahigh aspect ratio (Fig. 4a,b), it is inevitable that there are still openings where polysulfides can escape the structure and dissolve into the electrolyte, especially at the edges (Fig. 4a). These escaped polysulfides can cause serious changes in the battery system, and result in low Coulombic efficiency and subsequent capacity decay. Therefore, further modifications are required on the carbon structures to enhance the stability of the sulfur cathode. Inspired by the structure of a pie, which consists of thin skin and full filling, we come up with the idea of coating a thin layer of EFG on the surface of the free-standing LRC/S electrode. Our recent work shows that EFG not only enhances the conductivity of the sulfur-based cathode for better electron and ion transport, but also ensures strong adhesion of polar lithium polysulfides to the originally nonpolar carbon surface for more stable cycling^49^. EFG is synthesized by the same approach as previously reported^49^. Fourier transform-infrared spectra and X-ray photoelectron spectroscopy results of EFG confirm the strong chemical bonding between reduced graphene oxide (rGO) and amine functional groups (Supplementary Figs 7 and 8). Benefiting from the free-standing feature of the LRC/S paper electrode, EFG can be coated on the surface of the LRC/S paper electrode by a facile ‘dip and dry' approach, similar to a widely used method for fabrics dyeing in the textile industry. When the as-prepared LRC/S electrodes are dipped into the EFG ink, they can be quickly coated with EFG nanosheets. Subsequently, the ‘wet' LRC/S electrodes with EFG ink are dried in an oven at 70 °C for 2 h for solvent removal. By repeating this simple dip and dry process for 10 times, an unbroken EFG ‘crust' can be readily constructed outside the LRC/S electrode, forming the designed ‘pie' structure. Different from the black pyrolytic carbon colour of the LRC/S electrode, the LRC/S@EFG electrode displays a graphitic colour, while it still keeps the free-standing paper characteristic (Supplementary Fig. 9). Scanning electron microscope images of the LRC/S@EFG electrode show that a continuous wavy film, consisting of interconnected graphene sheets, closely adheres to the surface of the LRC/S electrode (Fig. 4c). The exposed external surface and opening ends of LRC/S nanofibers are all well wrapped by interconnected EFG nanosheets on both the front and side faces of the electrode (Fig. 4d,e). The cross-sectional image of a dissected electrode further confirms that the EFG layer is tightly attached on the exposed surface of the LRC/S electrode (Fig. 4f). It is noteworthy that the thickness of the EFG layer is <100 nm (Fig. 4g). Thermogravimetric analysis measurements verify that the content of EFG in the LRC/S@EFG electrode is about 15 wt% (Supplementary Fig. 10). The sulfur content in LRC/S@EFG is about 72.3 wt%, compared with 85.1 wt% in LRC/S. The areal mass loading of sulfur in the LRC/S@EFG electrode is still same with the LRC/S electrode. By measuring the weight and dimensions of the electrode, the density of the LRC/S@EFG electrode is calculated to be about 0.69 g cm^−3^.

### Electrochemical characterization

**Figure 5:**
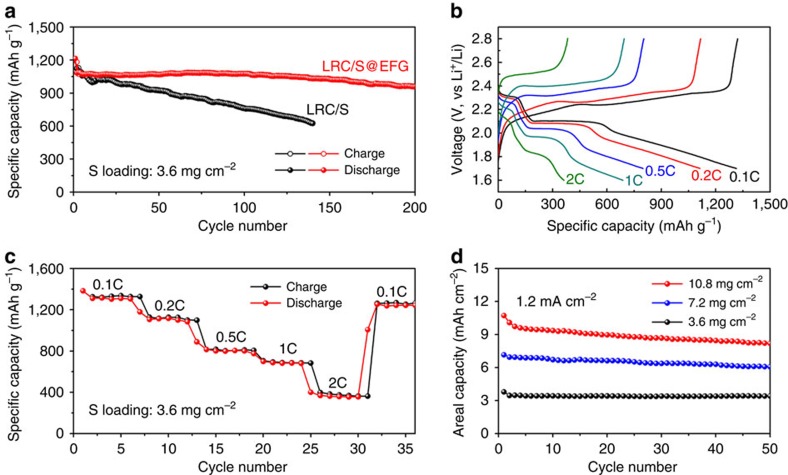
Electrochemical performances of LRC/S and LRC/S@EFG. (**a**) Cycle performance of LRC/S@EFG in comparison with LRC/S at a current density of 0.2 C. (**b**) Voltage profiles and (**c**) discharge capacities at various current densities from 0.1 to 2 C. (**d**) Areal capacities of layer-by-layer structured LRC/S@EFG electrodes during cycling at a current density of 1.2 mA cm^−2^.

To evaluate the electrochemical lithium storage performance, coin cells are fabricated directly using LRC/S@EFG electrodes as the cathode, and Li foil as the anode. Bare LRC/S electrodes are also assembled as cathode in coin cells for comparison. The typical sulfur mass loading of the LRC/S and LRC/S@EFG electrodes is about 3.6 mg cm^−2^, which is much higher than many slurry fabricated electrodes^53^. Figure 5a gives the cycling performance of both electrodes. At a current density of 0.2 C, the cells with LRC/S and LRC/S@EFG cathodes show similar initial discharge capacities of as high as 1,214 and 1,215 mAh g^−1^, corresponding to around 72% of the theoretical capacity of sulfur (1,675 mAh g^−1^), revealing the facile electronic/ionic transport and improved reaction kinetics enabled by the LRC framework. In the subsequent charge/discharge cycling, the LRC/S@EFG electrode shows much better stability with a reversible capacity of 950 mAh g^−1^ after 200 cycles, which corresponds to a small capacity decay of 0.1% per cycle. The discharge capacity of the LRC/S@EFG cathode actually increases slightly during the initial cycles, which is probably due to the gradual activation of sulfur in the smaller channels. In contrast, the cell with the LRC/S electrode suffers from steady capacity decay, showing a discharge capacity of 623 mAh g^−1^ by the 140th cycle, which should be caused by a rapid dissolution of polysulfide into the electrolyte.

To understand the improved cycling stability of LRC/S@EFG, the electrodes are further investigated after cycling. It can be seen that some lithium sulfides particles are deposited on the outside surfaces of LRC fibres (Supplementary Fig. 11a–d), indicating the dissolution of some polysulfides into the electrolyte from the LRC fibres during cycling test. The continuous dissolution and diffusion of polysulfides from the carbon framework may be the main reason for the poor cycle life of the LRC/S cells. On the other hand, the homogenous distribution of sulfur in the LRC/S@EFG framework after cycling (Supplementary Fig. 11e–l) suggests that the EFG layer can effectively allow the migration of Li^+^, while blocking the dissolution of polysulfides into the electrolyte, thus acting as an ion-selective membrane. The structural and functional differences between LRC/S and LRC/S@EFG are schematically illustrated (Supplementary Fig. 12).

Next, the rate performance of the LRC/S@EFG is investigated. Figure 5b shows the discharge/charge profiles under various C-rates from 0.1 to 2 C, corresponding areal current densities from 0.6 to 12 mA cm^−2^. The reversible discharge capacity is found to stabilize at ∼1,300 mAh g^−1^ (4.7 mAh cm^−2^; the values in the parentheses correspond to the areal capacities based on sulfur loading of 3.6 mg cm^−2^) at initially 0.1 C, and gradually decreases to 1,113 (4.0), 801 (2.9), 688 (2.5) and 363 mAh g^−1^ (1.3 mAh cm^−2^) as the current rate is increased to 0.2, 0.5, 1 and 2 C, respectively (Fig. 5b,c). When the current rate is reduced abruptly back to 0.1 C again, the LRC/S@EFG electrode is able to recover most of the original capacity, indicating outstanding stability and robustness of the pie-structured electrode. Based on the sulfur content of 72.3 wt%, electrode density of 0.69 g cm^−3^ and the specific capacity of ∼1,100 mAh g^–1^ (second cycle at 0.2 C), the capacity density of the LRC/S@EFG electrode reaches up to 549 mAh cm^−3^. The theoretical volumetric and specific energy densities of the Li–S cell with the LRC/S@EFG cathode are calculated as 867 Wh l^−1^ and 1,319 Wh kg^−1^, respectively (Supplementary Table 1).

We notice that the discharge–charge voltage profiles in this work are slightly different from that of conventional sulfur cathodes. Apart from two discharge plateaus at 2.3 and 2.1 V, there is a third slope-shaped discharge plateau in the voltage range of 2.0–1.7 V. This specific electrochemical behaviour is similar with the one observed from carbon nanotube/sulfur samples in the work by Wang *et al*.^54^, which were also prepared at elevated temperatures (300 or 500 °C). It is proposed that the initial S_8_ molecules might be converted to some smaller sulfur molecules, such as S_4_ and S_6_, due to the strong bonding energy between sulfur molecules and carbon defects^54^. Afterwards, the altered molecular structures probably influence the equilibrium potential of sulfur–lithium reactions, and result in slightly different electrochemical behaviour. The strong binding between sulfur molecules and carbon fibre can be revealed by Raman spectra of LRC and LRC/S (Supplemental Fig. 13)^55^. It is highly possible that some sulfur molecules have been intercalated into the lattice voids of amorphous carbon after high temperature (300 °C) treatment. Such tightly trapped sulfur molecules are speculated to be responsible for the third discharge plateau with slope. Another possible reason for this phenomenon is the crystal phase transition of sulfur caused by the elevated temperature treatment (400 °C)^36^. Kim's work shows that the monoclinic sulfur in hollow carbon nanowires (with average diameter of ∼75 nm) exhibits only a single discharge voltage plateau at the lower potential of ∼1.97 V (ref. 36). Summing up the discussions above, it is found that the sloping discharge plateau at lower potentials appears in the high aspect ratio shaped carbon–sulfur composites, which may result in much stronger binding energy between sulfur and carbon. The encouraging electrochemical performance indicates the promising use of these rationally designed sulfur–carbon cathodes for advanced Li–S batteries.

To explore the possibility of the LRC/S@EFG electrode for even higher sulfur mass loading, we have fabricated layer-by-layer structured LRC/S@EFG electrodes based on the concept of Manthiram's work^41^. Notably, by stacking the single-layered electrodes together, the sulfur mass loadings has been increased from 3.6 mg cm^−2^ to 7.2 mg cm^−2^ (two layers) and 10.8 mg cm^−2^ (three layers). At a current density of 1.2 mA cm^−2^, the cells with different (three, two and one) layers of LRC/S@EFG electrodes deliver initial discharge capacities of 10.7 (993), 7.2 (995) and 3.8 mAh cm^−2^ (1,054 mAh g^−1^), respectively, and show good capacity retention (Fig. 5d, Supplementary Fig. 14). These results further indicate that the pie-structured LRC/S@EFG electrode not only endows excellent conductivity of the entire sulfur-based cathode, but also effectively confines the soluble polysulfide within the carbon framework.

## Discussion

The remarkable improvement on battery performance and energy density of the ‘pie'-structured LRC/S@EFG electrode benefits from the combined effect of the highly efficient LRC/S as ‘filling' and the robust EFG layer as ‘crust'. First, the structure of LRC nanofibers is well suited to be applied as a sulfur host with two main advantages: (1) with an average channel diameter of 60 nm, LRC nanofibers provide sufficient hollow space for sulfur loading and 85 wt% of sulfur can be encapsulated; (2) comparing with the disordered void structures, the highly parallel channels forming an excellent conductive framework at nanoscale affords efficient ion/electron accessibility of the active material^20^. With close contact of the confined sulfur and intermediate lithium sulfides, each channel inside LRC nanofibers serves as a nanoscale electrochemical reaction chamber, leading to a much more complete redox reaction of the active material. Second, the external EFG ‘crust' possesses the strong capability for binding polysulfides, serving a function similar to the interlayers proposed by Manthiram's group^15^. Unlike the interlayer that is located between the cathode and the separator, the nanoscale EFG layer in our work completely wraps the internal LRC/S structure. Therefore, it effectively prevents polysulfides from migrating in all directions, especially at the side of the electrode and results in substantial improvement for the cycling stability of Li–S batteries. The weight fraction of EFG layer in the final electrode is only 15 wt%, with little adverse impact on the sulfur mass ratio and the cell energy density.

In summary, a pie-structured LRC/S@EFG electrode with free-standing LRC/S as ‘filling' and interconnected EFG layer as ‘crust' has been developed with an excellent balance between electrochemical performance and areal energy density. By electrospinning a mixed polymer precursor of PAN/PS, 3D networks of multichannel LRC nanofibers are successfully fabricated with controllable channel structure in each fibre. The nanofiber mat is applied as a free-standing and binder-free electrode with 85.1 wt% sulfur loading. Through a simple dip and dry method, a thin EFG layer is closely wrapped outside the LRC/S electrode to prevent the diffusion of polysulfides from cathode structure and enhance the cycling stability of the LRC/S@EFG electrode. With combined effects of LRC/S and EFG, the final sulfur cathode is able to deliver high capacity of 1,314 mAh g^−1^ (4.7 mAh cm^−2^) at 0.1 C (0.6 mA cm^−2^) accompanied with excellent cycling stability. The areal capacity could be further boosted to more than 8 mAh cm^−2^ by stacking three layers of LRC/S@EFG electrodes. We believe that these electrospun multichannel carbon nanofibers (‘filling') and the facile method for entire electrode dip coating with EFG (‘crust') may have some real impact on the development of practical high-performance Li–S batteries.

## Methods

### Synthesis of lotus LRC nanofibers textile

The precursor solution for electrospinning was prepared by dissolving PS (0.5, 1.0, 2.5 or 5.0 g for different samples) and PAN (5.0 g) in N,N-dimethylformamide (50 ml) with vigorous stirring at 60 °C overnight. Then PAN/PS composite nanofibers were electrospun on aluminium foil collector from the precursor solution. The distance between the syringe and the collector was fixed at 15 cm, and the voltage of 15 kV was applied with a flow rate of 1 ml h^−1^. Finally, the LRC mat was obtained by carbonization of the PAN/PS nanofibers film at 800 °C for 3 h with heating rate of 3 °C min^−1^ under argon atmosphere.

### Synthesis of EFG ink

EFG was prepared as reported^49^. For a typical synthesis, graphene oxide was first prepared via a modified Hummers' method^56^, and dispersed in deionized water (0.5 mg ml^−1^). Then, 240 μl of EDA was mixed with 300 ml of graphene oxide suspension in a sealed glass vessel, and heated at 75 °C for 6 h with continuous stirring. After that, the EFG was washed thoroughly with deionized water by centrifugation, and then re-dispersed (0.5 mg ml^−1^).

### Synthesis of the LRC/S@EFG electrode

Several pieces of LRC mat were sealed with commercial sulfur powder (1:10, w/w) in a stainless steel vessel under argon atmosphere protection, then heated at 300 °C for 12 h in a quartz tube furnace under argon atmosphere. After the heating treatment, free-standing LRC/S electrodes were obtained. After cutting, LRC/S electrodes were completely dipped into the EFG ink and immediately taken out, and the ‘wet' electrodes were then placed into an oven at 70 °C for 2 h to remove the solvent. This simple ‘dip and dry' process was repeated for 10 times to obtain a robust coating layer of EFG adhering on the surface of the LRC/S electrode.

### Materials characterization

The structures and morphologies of the samples were characterized with field-emission scanning electron microscopy (JEOL, JSM-6700 F) and TEM (JEOL, JEM-2100 F). Elemental mapping and linear scanning were performed on the energy dispersive X-ray spectroscopy attached to the JEM-2100 F. The material phase information was examined by X-ray diffraction (Bruker D2 Phaser X-Ray Diffractometer with Cu Kα radiation *λ*=1.5406 Å). Sulfur content was determined by thermogravimetric analysis (Shimadzu DRG-60) in N_2_ flow.

### Electrochemical measurements

Both LRC/S and LRC/S@EFG papers were directly used as working electrodes after vacuum drying at 60 °C overnight. The areal mass loading of sulfur (3.6±0.2 mg cm^−2^ for single layer) was controlled by the thickness of the electrodes. CR2032 coin cells were assembled in an Ar-filled glovebox with lithium metal as anode and Celgard 2300 membrane as separator. The electrolyte was 1 mol l^−1^ lithium bis(trifluoromethanesulfonyl)imide in 1, 3-dioxolane and dimethoxymethane (v/v=1:1) with 0.2 mol l^−1^ LiNO_3_. The electrolyte volume is controlled to be 20 μl per 1 mg of the electrode. The galvanostatic charge/discharge measurements were performed in a voltage cutoff window of 1.7–2.8 V using a NEWARE battery tester. All the capacity values were based on the mass of sulfur.

## Additional information

**How to cite this article:** Li, Z. *et al*. Pie-like electrode design for high-energy density lithium–sulfur batteries. *Nat. Commun.* 6:8850 doi: 10.1038/ncomms9850 (2015).

## Supplementary Material

Supplementary Information Supplementary Figures 1-14, Supplementary Table 1 and Supplementary References
