# Supplementary material for: Pie-like electrode design for high-energy density lithium–sulfur batteries
Source: Nat Commun. 2015 Nov 26;6:8850. doi: 10.1038/ncomms9850 (PMC4674769; doi:10.1038/ncomms9850)
Supplement: Supplementary Information — Supplementary Figures 1-14, Supplementary Table 1 and Supplementary References [file ncomms9850-s1.pdf]

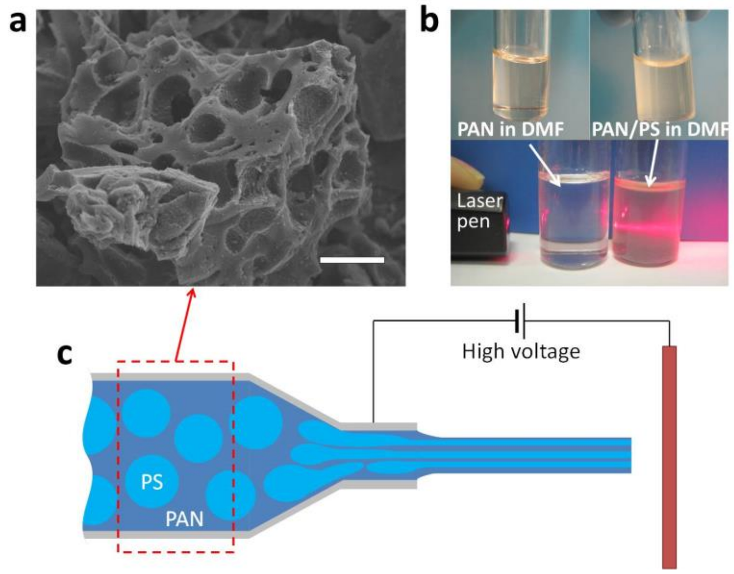

Supplementary Figure 1. **The formation mechanism of LRC fibers.** (a) SEM image of the directly carbonized mixture of PAN/PS (1:0.5, weight ratio). (b) Digital photos of the DMF solution containing PAN and the mixture of PAN/PS (1:0.5, weight ratio). (c) Schematic illustration of the formation mechanism of multichannel structured LRC fibers. Scale bar of (a) is 10 μm. Precursor solution containing the mixture of PAN/PS (1:0.5, weight ratio) was dried and carbonized at 800 ℃ under N_2 to exhibit the distribution status of PS. After the pyrolysis treatment, PS decomposed, and PAN is converted into carbon. The micron and sub-micron scaled pores in the carbon particle correspond to the PS occupied space (Supplementary Fig. 1a), revealing that, in the precursor solution, PS is not as well dissolved in DMF as PAN, but forms a micro emulsion, which can be further confirmed by the light scattering in the PAN/PS solution (Supplementary Fig. 1b). The micro emulsion of PS could be stretched into parallel nanowires in the PAN fibers by electrospinning, and then decomposed to generate nanochannels during the pyrolysis process (Supplementary Fig. 1c).

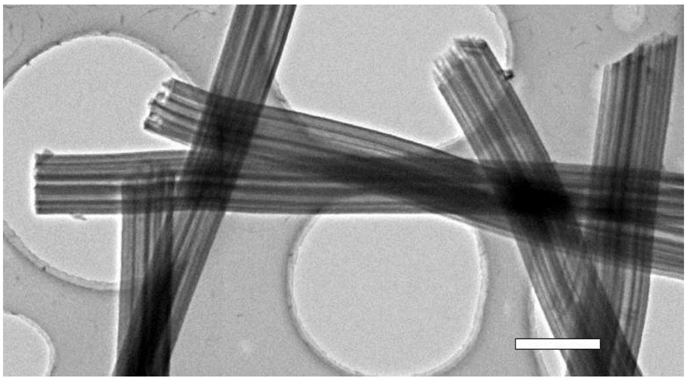

Supplementary Figure 2. **Characterization of LRC nanofibers.** TEM image of LRC nanofibers derived from pyrolysis of PAN/PS (1 : 0.5) nanofibers. Scale bar, 1 μm.

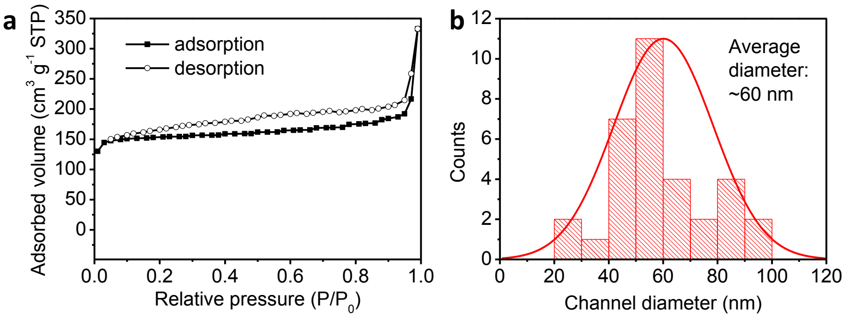

Supplementary Figure 3. Channel structural characteristics of LRC nanofibers. (a) N_2 sorption isotherms. (b) Channel size distribution from TEM images.

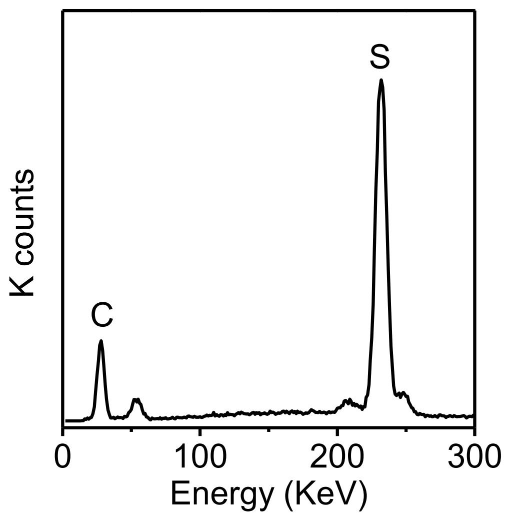

Supplementary Figure 4. **EDX spectrum of the LRC/S electrode.**

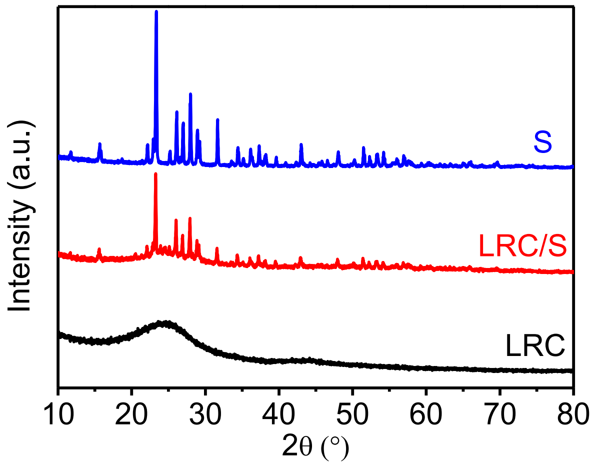

Supplementary Figure 5. **XRD patterns of S, LRC/S and LRC.**

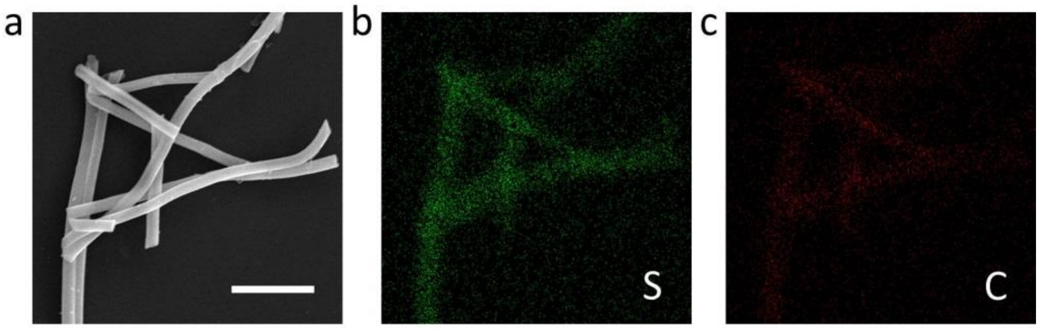

Supplementary Figure 6. **Characterization of LRC/S nanofibers.** (a) SEM image and corresponding elemental mappings of (b) sulphur and (c) carbon. Scale bar, 5 μm.

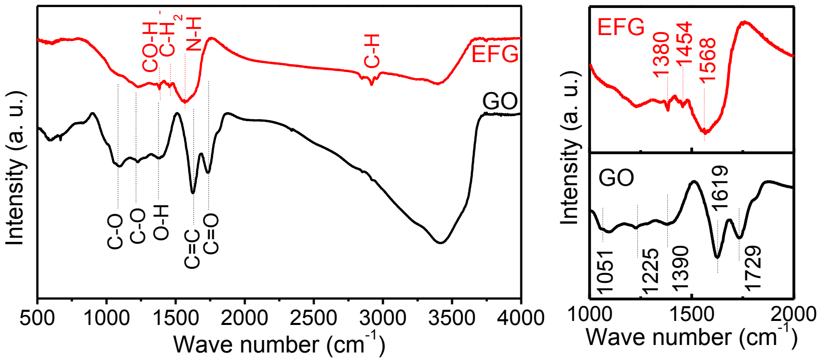

Supplementary Figure 7. FT-IR analysis. FT-IR spectra of GO and EFG. In the spectrum of EFG, the adsorption peaks of C=O, –OH and C–O bonds in teh GO sample are greatly suppressed, while new peaks arise at 1568, 1150–1465 cm^{-1}, that can be assigned to the antisymmetric C–N stretching vibrations coupled with out-of-plane NH_2 and NH modes, as well as the N–H stretching vibrations[1, 2].

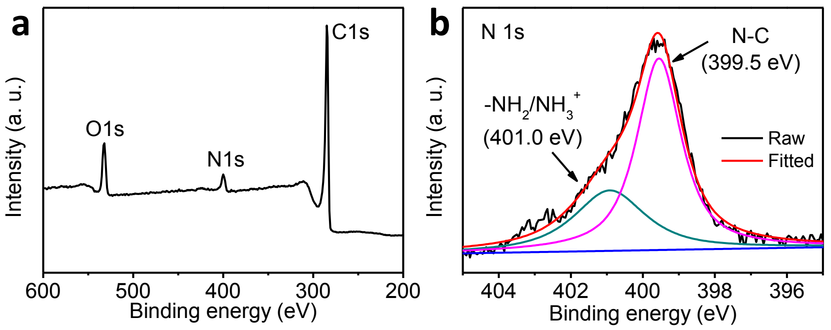

Supplementary Figure 8. XPS analysis. (a) Survey XPS spectrum of EFG, and (b) N 1s XPS spectrum. The deconvoluted N 1s spectra of as-prepared EFG sample show the domination of N-C binding at 399.5 eV and presence of $–NH_2/NH_3^+$ groups at 401.0 eV, which further confirms the chemical bonding between rGO and EDA moiety.

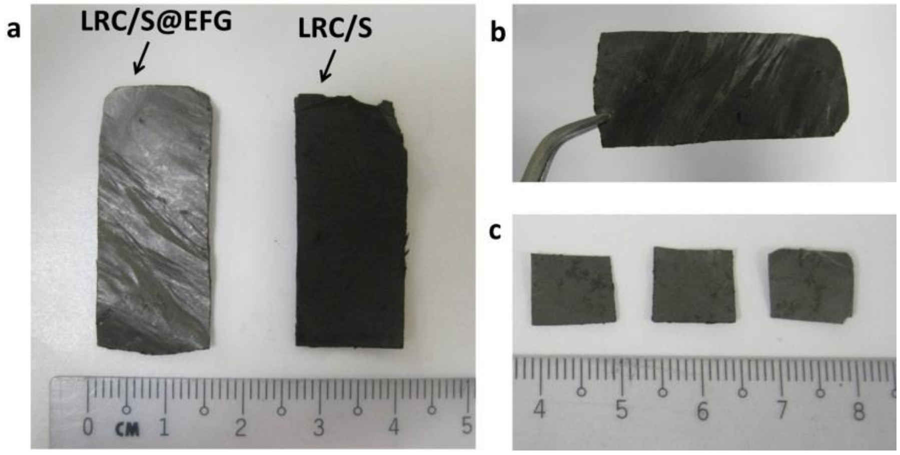

Supplementary Figure 9. **Digital photos of the as-prepared electrode.** (a) Comparison of the visible colors between the LRC/S@EFG electrode and the LRC/S electrode. (b) A typical digital photo showing the free-standing characteristic of the LRC/S@EFG electrode. (c) The LRC/S@EFG electrodes with proper size for coin cell tests.

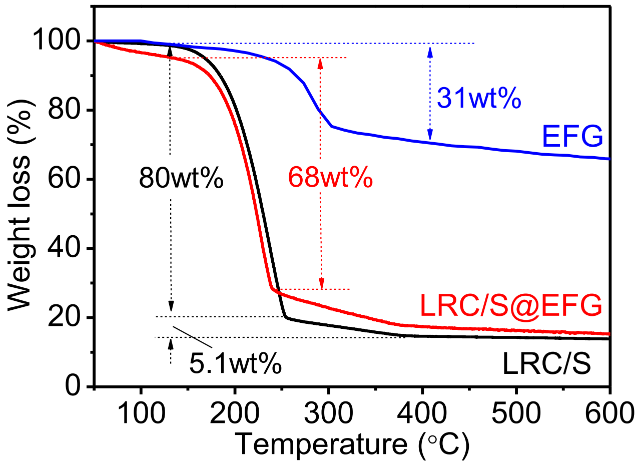

Supplementary Figure 10. **Thermogravimetric analysis of LRC/S, LRC/S@EFG and EFG.** All the tests were conducted with a heating rate of 10 ℃ min^{-1} in N_2 atmosphere. To gain accurate mass ratio data, the samples for TGA tests were prepared by grinding a piece of free-standing electrode into powder, and take ~1 mg of powder for each measurement. Because EFG has weight loss in the temperature range from 240 to 400 ℃, the sulphur content of LRC/S@EFG cannot be measured directly on the TGA curve. It is found that there are two sections of the weight loss for sulphur (80 wt% and 5.1 wt%) in LRC/S, so the total sulphur content of LRC/S@EFG can be estimated based on the first weigh loss section of sulphur (68 wt%) as: 68 wt% × (1 + 5.1 wt% / 80 wt%) = 72.3 wt%. Then, the LRC content of LRC/S@EFG can be estimated as: 72.3 wt% × (1 – 85.1 wt%) / 85.1 wt% = 12.7 wt%. Finally, the content of EFG in LRC/S@EFG can be estimated as: 100 wt% –72.3 wt% – 12.7 wt% = 15 wt%.

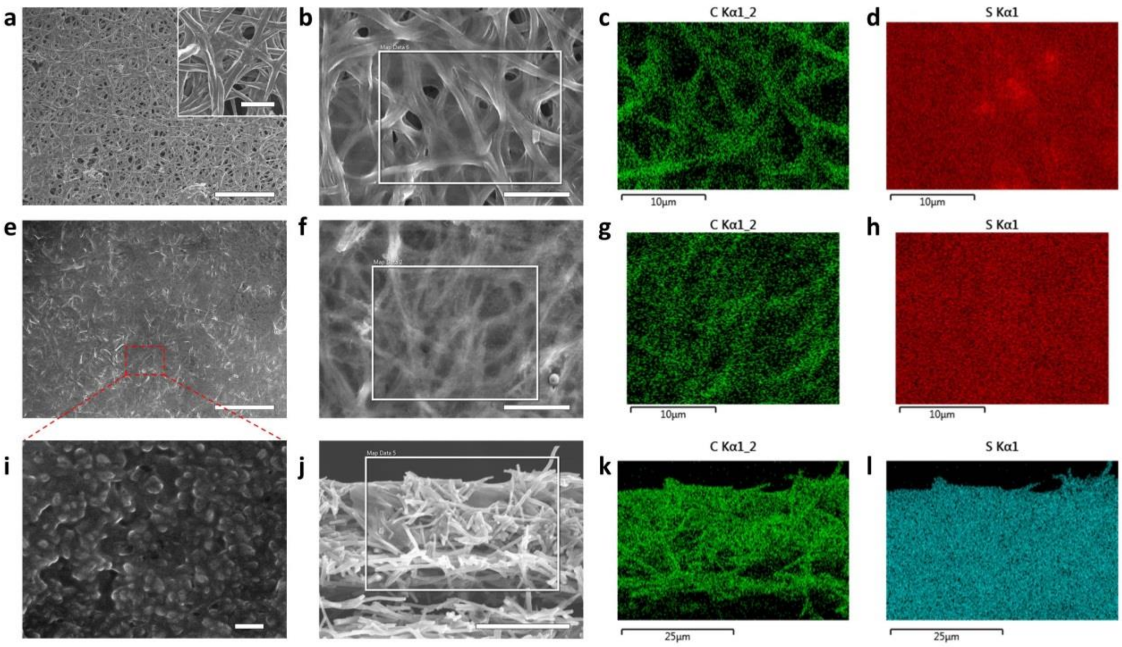

Supplementary Figure 11. **Characterization of the cycled LRC/S and LRC/S@EFG electrodes.** SEM images and corresponding EDX elemental mappings of (a-d) the LRC/S electrode and (e-l) the LRC/S@EFG electrode after 50 cycles at 0.2 C. The cells were disassembled at 3.0 V in an argon filled glove box, and the electrode films were washed with DOL solvent for several times before taken out for characterization. Scale bars, 50 μm (a, e), 10 μm (b, c, d, f, g, h), 200 nm (i), 25 μm (j, k, l), 5 μm (inset of a).

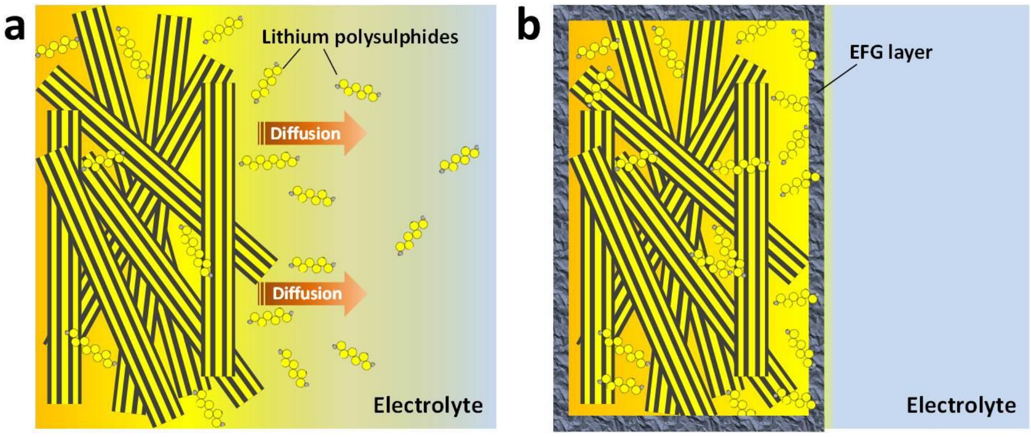

Supplementary Figure 12. Schematic illustration of the LRC/S and LRC/S@EFG electrodes during the cycling test. Schematic electrode structures of (a) LRC/S and (b) LRC/S@EFG. Since polysulphides could easily diffuse into electrolyte, the LRC/S electrode suffered from active material lose and continuous capacity fading. Benefitting from the efficient blocking of polysulphides by the EFG layer, the LRC/S@EFG electrode is able to maintain stable reversible capacity.

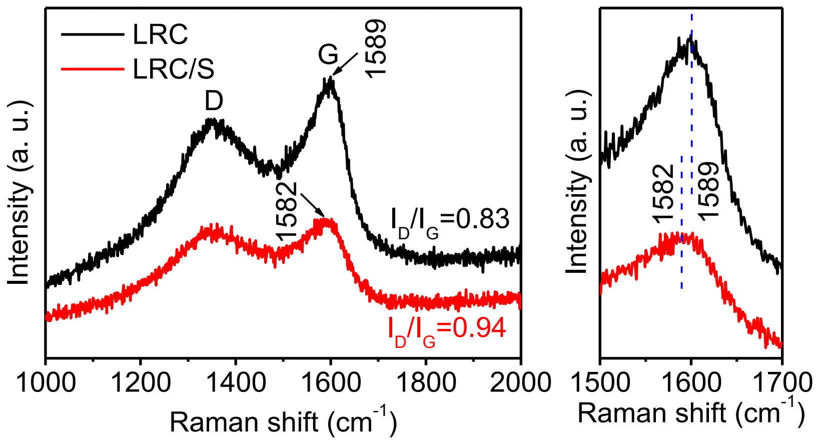

Supplementary Figure 13. **Raman spectra of LRC and LRC/S.** Two prominent peaks at 1338 and 1582 cm^{-1} of the LRC/S composite correspond to the D and G bands of carbon substrate respectively. The G band shift in carbon-based composites relates to the charge transfer between the carbon and other dopants[3]. Therefore, the observed shift by 7 cm^{-1} from 1589 (LRC) to 1582 cm^{-1} (LRC/S) indicates the presence of a charge transfer from carbon substrate to sulphur molecules. The Raman shift in the LRC/S composite could be induced by the doping effect and/or bonding formation, revealing the strong binding between sulphur and carbon.

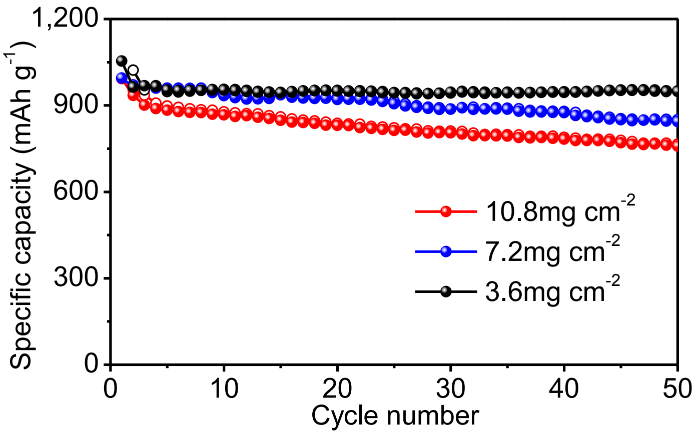

Supplementary Figure 14. **Cycling performance of Li-S cells with different number (3, 2, 1) of layers of LRC/S@EFG electrode.** All cells are tested at a current density of 1.2 mA cm^{-2}, corresponding to varied C-rates of 0.066, 0.1 and 0.2 C for the electrodes with sulphur mass loadings of 10.8, 7.2 and 3.6 mg cm^{-2}, respectively.

Supplementary Table 1. Theoretical energy densities of Li-S cells

Cathode/Anode	Cathode Capacity (mAh/g)	Active material content	Cathode density (g/cm^3)	Anode Capacity (mAh/g)	Anode density (g/cm^3)	Voltage difference (V)	Energy density* (Wh/L)	Energy density* (Wh/kg)
S *vs.* Li (Theoretical)	1675	100%	2.07	3860	0.534	2.2	2844	2600
LRC/S@EFG *vs.* Li (This work)	~1100 (at 0.2C)	72.3%	0.69	3860	0.534	2.0	867	1319

* The energy densities are calculated based on only cathode and anode by the same method as reported[4], excluding electrolyte, separator and other additive materials.

Supplementary References

1. Hu, H., Zhao, Z., Wan, W., Gogotsi, Y. & Qiu, J. Ultralight and highly compressible graphene aerogels. *Adv. Mater.* **25**, 2219-23 (2013).

2. Compton, O.C., Dikin, D.A., Putz, K.W., Brinson, L.C. & Nguyen, S.T. Electrically conductive "alkylated" graphene paper via chemical reduction of amine-functionalized graphene oxide paper. *Adv. Mater.* **22**, 892-6 (2010).

3. Rao, A.M., Eklund, P.C., Bandow, S., Thess, A. & Smalley, R.E. Evidence for charge transfer in doped carbon nanotube bundles from Raman scattering. *Nature* **388**, 257-9 (1997).

4. Yang, Y., McDowell, M.T., Jackson, A., Cha, J.J., Hong, S.S. & Cui, Y. New nanostructured Li_2S/silicon rechargeable battery with high specific energy. *Nano Lett.* **10**, 1486-91 (2010).
